# Identification of Potential Small Molecule Allosteric Modulator Sites on IL-1R1 Ectodomain Using Accelerated Conformational Sampling Method

**DOI:** 10.1371/journal.pone.0118671

**Published:** 2015-02-23

**Authors:** Chao-Yie Yang

**Affiliations:** Department of Internal Medicine, Hematology and Oncology Division, University of Michigan, Ann Arbor, Michigan, United States of America; Faculdade de Ciências da Universidade de Lisboa, PORTUGAL

## Abstract

The interleukin-1 receptor (IL-1R) is the founding member of the interleukin 1 receptor family which activates innate immune response by its binding to cytokines. Reports showed dysregulation of cytokine production leads to aberrant immune cells activation which contributes to auto-inflammatory disorders and diseases. Current therapeutic strategies focus on utilizing antibodies or chimeric cytokine biologics. The large protein-protein interaction interface between cytokine receptor and cytokine poses a challenge in identifying binding sites for small molecule inhibitor development. Based on the significant conformational change of IL-1R type 1 (IL-1R1) ectodomain upon binding to different ligands observed in crystal structures, we hypothesized that transient small molecule binding sites may exist when IL-1R1 undergoes conformational transition and thus suitable for inhibitor development. Here, we employed accelerated molecular dynamics (MD) simulation to efficiently sample conformational space of IL-1R1 ectodomain. Representative IL-1R1 ectodomain conformations determined from the hierarchy cluster analysis were analyzed by the SiteMap program which leads to identify small molecule binding sites at the protein-protein interaction interface and allosteric modulator locations. The cosolvent mapping analysis using phenol as the probe molecule further confirms the allosteric modulator site as a binding hotspot. Eight highest ranked fragment molecules identified from *in silico* screening at the modulator site were evaluated by MD simulations. Four of them restricted the IL-1R1 dynamical motion to inactive conformational space. The strategy from this study, subject to *in vitro* experimental validation, can be useful to identify small molecule compounds targeting the allosteric modulator sites of IL-1R and prevent IL-1R from binding to cytokine by trapping IL-1R in inactive conformations.

## Introduction

The interleukin-1 (IL-1) family of ligands and receptors are important regulators of innate inflammatory and immune responses[1]. Currently, eleven IL-1 ligands (or cytokines) and receptors were identified respectively [2]. These IL-1 ligands function as agonists and antagonists to IL-1 signaling by binding to IL-1 receptors including ten single pass transmembrane proteins and IL-18BP which lacks transmembrane domain[3]. Regulation of inflammatory signaling via IL-1 family of receptors can be mediated either by its binding to the activating cytokines or antagonistic ligands such as IL-1Ra which inhibit downstream signaling [4]. Some members of the IL-1 receptors are decoy receptors such as IL-1 receptor type 2 (IL-1R2) which lacks the cytoplasmic element to recruit downstream effector proteins. Additional level of regulation is controlled via the cleavage of the ectodomain of IL-1 receptors such as that found in IL-1 receptor type 1 (denoted sIL-1R1)[5] or the alternatively spliced soluble Suppressor of Tumorigenicity 2 (sST2) protein[6]. sIL-1R1 and sST2 have only the ectodomain of their respective receptors, IL-1R1 and ST2. They circulate in the extracellular milieu to modulate their respective binding cytokines concentrations and attenuate the inflammatory responses mediated by these ligands [5,7,8].

Roles of many members in the IL-1 family play in inflammatory diseases and auto-inflammatory diseases or disorders have been reported[9]. In one case, loss of function homozygous mutation in the endogenous IL-1 receptor antagonist (IL-1Ra) has been identified in infants with early death due to overwhelming systemic inflammation found in skins, joints and bones[10]. Contribution of the IL-1 family of proteins to cardiovascular diseases, Type-2 diabetes, rheumatoid arthritis and chronic inflammatory diseases have also been reported[9]. Current available therapy for treating IL-1 family related diseases is limited to Canakinumab, Gevokizumab and LY2189102 which are mainly anti-IL-1β monoclonal antibodies[9]. No small orally available drugs are available. In recent years, the expanding roles of the member proteins in IL-1 family play in multiple diseases have been discovered [2,11]. One example is that the elevated level of soluble ST2 in the plasma of patients receiving allogeneic hematopoietic stem cell transplantation predicted their graft-versus-host disease (GVHD) mortality [12,13]. The prognostic value of soluble ST2 was attributed to its role as a decoy receptor of IL-33. Binding of sST2 and IL-33 decreases the level of unbound IL-33 in plasma which drives type-2 T cells towards a type-1 T cell response during transplantation. Thus, there are needs to discover and develop new therapy targeting this important cytokine family of proteins for treating inflammatory and immune-mediated diseases.

Currently, FDA approved specific IL-1 targeting therapies are monoclonal antibodies. Orally available small molecule drugs have advantages in several aspects including ease of administration, cost effectiveness and flexible dosage schedule design. Given the chronic nature of the inflammatory disorders and diseases associated with IL-1 family of proteins[14], efficacious orally available drugs will benefit patients in their long-term treatment. To develop small molecule inhibitors for the IL-1 family of proteins, we focus on targeting the ectodomain of IL-1 receptor type 1 (IL-1R1) protein, the first cytokine receptor in the family discovered[15], in this work. Binding of IL-1R1 with IL-1α or IL-1β leads to subsequent recruitment of IL-1RAcP at the cell membrane [16] and downstream cytosolic NF-κB activation. A small molecule probe (NP1) was reported recently to inhibit the association between IL-1R and TLR4 and the downstream NF-κB activation[17]. However, it was suggested to bind to the cytosolic component of the cytokine receptor protein complex without clear indication of the protein target.

Available crystal structures of IL-1R1 indicated that IL-1R1 binds with IL-1β using three domains (D1,D2 and D3) and their contact interface (4180.5 Å^2^) is much larger than those in typical small molecule binding sites (1500–3000 Å^2^)[18]. While IL-1R1 binds with IL-1Ra involving small conformational changes of the D3 domain [19], significant changes were found when IL-1R1 binds with a peptide antagonist (AF10847)[20]. We hypothesized that the flexibility of IL-1R1 ectodomain observed from crystal structures may indicate the existences of multiple conformations that can potentially be exploited by small molecule inhibitors development. To investigate this inhibitor development strategy, we employed computational methods to first sample extensively IL-1R1 conformations based on accelerated molecular dynamics simulation algorithm[21]. Selected represented conformations determined by the hierarchy cluster method were analyzed by the Sitemap program[22] to identify “druggable” small molecule binding sites. Potential small molecule binding sites, denoted as allosteric modulator sites, not at the cytokine receptor/cytokine interaction interface were determined. The druggable binding sites were further assessed as binding hotspots via our recently developed cosolvent mapping method [23–25] which incorporated binding site flexibility. An *in silico* screening of a commercial fragment library was then used to target one modulator site in an unreported inactive IL-1R1 conformation. From the eight top-ranked compounds, we found four compounds bound to the allosteric modulator site for extended period of time and restricted the IL-1R1 dynamical motion to an inactive conformational space during the 8 ns MD simulations. The approach described here can be an attractive strategy to discover small molecule inhibitors targeting the challenging cytokine receptor proteins.

## Materials and Methods

### Protein structures of IL-1R1 used in this study

Five crystal structures of protein complexes containing IL-1R1 ectodomain have been deposited in the protein databank (PDB entries: 4DEP[26], 4GAF[19], 3O4O[27], 1G0Y[20], 1IRA[28], 1ITB[29]). They include IL-1R1/IL-1β (1ITB), IL-1R1/IL-1β/IL-1RAcP (4DEP), IL-1R1/IL-1Ra (1IRA), IL-1R1/EBI-005 (4GAF) and IL-1R1/AF10847 (1G0Y). EBI-005 is an IL-1β chimera and binds more potently to IL-1R1 than IL-1β[19]. The backbone structures of IL-1R1 underwent minor changes in IL-1R1/IL-1β, IL-1R1/IL-1β/IL-1RAcP and IL-1R1/EBI-005 in which the root-mean-square deviations between them are 0.8 Å. We selected the IL-1R1 in the IL-1R1/EBI-005 as the initial conformation for our conformational sampling simulation because of its highest structural resolution (2.15Å) and more amino acid structures resolved at the N-terminus. This IL-1R1 structure contains sequence D21-P330 of the extracellular domain (or ectodomain) with no missing atoms between D21 and P330. Among the crystal structures, four asparagines were glycosylated except the structure in 1ITB[29]. The glycosylated asparagines do not interact directly with the endogenous cytokine ligands. During our simulations, the sugar molecules on IL-1R1 were removed. Simulations of the IL-1Ra and AF10847-bound IL-1R1 conformations were also performed using the sequences of D21-V331 and C23-T332 respectively for comparison purpose. Five disulfide bonds in IL-1R1 were maintained in all simulations.

### MD simulation setup

In preparation of the simulations, we have used the MOE program[30] to determine the protonation state of ionizable groups on IL-1R1 under the standard physiological condition. PMEMD from Amber (version 12)[31] was used for molecular dynamics simulations. The Amber 99SB force field parameters[32] were used for the amino acids. To prepare the topology and coordinate files, counter ions were added to neutralize the charges in IL-1R1 before it was placed in a 13Å octahedral box of water. The TIP3P[33] water model was used. A 3000-step minimization (steps 1–1000 using conjugated gradient followed by 2000 steps steepest decent) was first carried out. After minimization, a 500 ps constant volume and constant temperature (NVT) simulation was performed to raise the temperature of the system to 298K while constraining backbone atoms with a 5 kcal/mol/Å^2^ force constant with reference to the crystal structure. A second 200 ps constant pressure and constant temperature (NPT) simulation at 298 K was performed while constraining backbone atoms with a 2 kcal/mol/Å^2^ force constant with reference to the crystal structure. The system was then equilibrated for 1 ns at 298K without any constraints followed by the production run. All the MD simulations were in the isobaric isothermal (NPT, T = 298K and P = 1 atm) ensemble. The SHAKE[34] algorithm was used to fix bonds involving hydrogen. The PME method[35] was used and the non-bonded cutoff distance was set at 10Å. The time step was 2 fs, and neighboring pairs list was updated every 20 steps.

For performing accelerated MD simulations, a 2 ns conventional MD (no modification of the potential energy function) was conducted to determine the average values of the potential energy for the total system (V_total_) and the dihedral angles energy of the proteins (V_dih_). The threshold potential energy was defined as V_total_ + N_atoms_/5 (N_atoms_: total number of atoms in the system). Three threshold potential energies of the dihedral angle motion were used called AMD1, AMD2 and AMD3 with V_dih_ + (number of residues) times 3.5, 4.2 and 4.9 respectively. These parameters were taken as suggested by previous works[21]. The V_total_ and V_dih_ parameters used in each machine were based on the averaged potential energy values calculated respectively. A total simulations time of 118 ns were collected from 2 ns equilibrium production run, 40 ns run of AMD1 parameters, 10 ns run of AMD2 parameters, 10 ns run of AMD3 parameters performed at the GORDON cluster supported by XSEDE, 30 ns run of AMD1 parameters in a local GPU machine and 26 ns run of AMD1 in an eight cores local machine. The lengths of the simulations performed at the GORDON cluster were limited by the computation times allocated to this work by XSEDE. Additional simulation runs performed at the local machines were included to ensure the coverage of the conformational space guided by the projections of conformations to the principal component spaces discussed later in the Methods section.

The procedures of performing cosolvent mapping simulation and analysis have been reported previously [23–25]. In this work, we used phenol as the probe molecule because the phenol group is frequently found in the fragment screening against proteins involved in protein-protein interaction[36]. Cosolvent mapping analyses were done using the trajectories of 4 ns cosolvent MD simulations.

Force field parameters of the small molecules were derived using the Antechamber module in the Amber program suite. The protocol for generating the point charge parameters is as follows: The docked pose of each molecule was minimized at the RHF level using a 6–31G* basis set with Gaussian09 [37]. The electrostatic field potential calculated from Gaussian09 was used to derive the point charges at each atom site based on the RESP fitting procedure[38].

### Principal component analysis and hierarchy cluster analysis of the conformations

Five crystal structures of IL-1R1 bound to different ligands were used in the principal component analysis (PCA) implemented in the Bio3D version 2.0[39]. In the PCA, amino acid sequences of the crystal structures were aligned and gaps detected based on MUSCLE[40]. Cα atoms coordinates of non-gap amino acids in the sequence alignments were first superimposed. Those with largest displacements were iteratively removed to reach a core set of amino acids to represent the structurally “static” amino acids among the variations of conformations analyzed. Based on the alignment of the core set of amino acids, the averaged displacements of all non-gap amino acids were calculated to construct the covariance matrix. Diagonalization of the matrix gives eigenvectors and eigenvalues. The eigenvectors are referred as principal components (PCs) and the coefficients of the eigenvectors characterize the collective atomic displacements for each principle component. The eigenvalues of the matrix gives the contribution of each eigenvector to the overall displacements in the covariance matrix. Such an analysis can be considered as the linear transformation of the coordinate system from Cartesian bases to collective movement bases. The PC gave the physical representation of collection motion in the protein dynamics because the Cα atoms were connected (or correlated) via backbone peptide bonds. Detailed procedures can be found in the Bio3d website. Snapshots of conformations obtained from each MD simulation were then first aligned to the initial conformation and the conformational changes in the simulated trajectory were mapped to the associated principal components shown in figures. The PCA using the five crystal structures showed the first three principal components contributed 94, 4.6 and 0.7% to the overall structural displacements. Mapping of conformations from the trajectory to two principal components informed a two dimensional subspace characterization of the conformational changes and/or transition.

For hierarchy cluster analysis, a second PCA of 1180 aMD generated IL-1R1 conformations taken at every 100 ps from the 118 ns trajectory were first performed without including the crystal structures. For this hierarchy cluster analysis, we used the first 38 PCs, which accounted for 90% of backbone structural variation of the 1180 IL-1R1 conformations. In the cluster analysis, the Euclidean distances between the 38 PCs were used to construct the dendrogram as implemented in the hclust function of the R program[41]. By using a cutoff value of 40 Å at the dendrogram, we obtained 25 cluster groups. The conformation closest to the center of each cluster group was used to represent each cluster group, also called representative conformation.

### Sitemap Analysis

Three crystal structures (chain A from 4GAF[19], chain R from 1IRA[28], chain B from 1G0Y[20]) and twenty five representative conformations of IL-1R1 determined from the hierarchy cluster analysis were used in the Sitemap program [22] to identify potential small molecule binding sites in each conformation. Each conformation was first processed in the Glide program[42] from the Maestro 9.7 program suite (Release 2014-1)[42] followed by the Sitemap analysis using default parameters. Up to five binding sites were saved. Among the twenty five MD generated conformations, only conformation **399** gave four binding sites. Druggability index of each binding site was based on the Dscore value in the Sitemap program.

### *In silico* screening and the fragment library

Conformation **289** was used in the *in silico* fragment library screening. The virtual screening workflow procedure in the Glide program was adopted in the screening calculation. Val117 of IL-1R1 (P2 site) was selected as the center of the docking site and the inner and outer rectangular box sizes were set to 10 and 30 Å respectively in the grid generation. The Maybridge fragment library satisfied the rule of three[43] were obtained from the Maybridge website with a total of 2783 compounds. Structures of the Maybridge fragment library were prepared using the Ligprep program in the Maestro program suite using the default setting. Two compounds (both adamantine derivatives) failed in the Ligprep program and were fixed manually. A total of 4409 compound conformations were obtained and used in the *in silico* screening calculations. The poses of the top eight ranked compounds based on the XP docking function from the *in silico* screening were inspected and used in the subsequent MD simulations and analyses.

## Results and Discussions

### Crystal structures between IL-1R1 and different ligands

**Fig 1:**
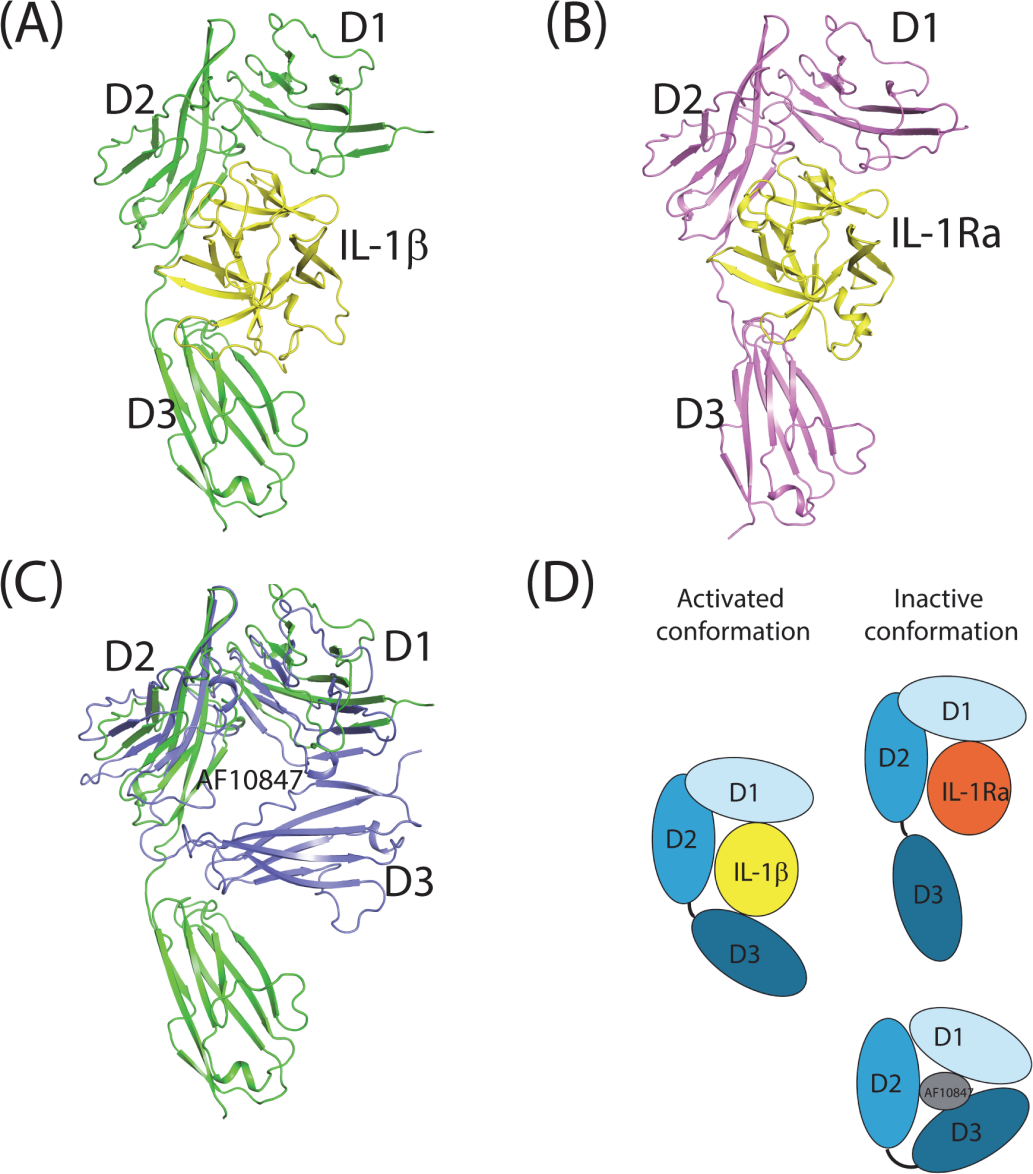
Crystal structures of IL-1R1 with three different ligands. (A) IL-1R1/IL-1β (PDB entry: 4DEF), (B) IL-1R1/IL-1Ra (PDB entry: 1IRA), and (C) IL-1R1/AF10847 (PDB entry: 1G0Y) superimposed with IL-1R1 from IL-1R1/IL-1β. (D) Cartoon depiction of activated and inactive conformations of IL-1R1 with three types of ligands, i.e. IL-1β, IL-1Ra and AF10847. AF10847 was not shown for clarity.

Three different conformations of IL-1R1 ectodomain have been determined via X-ray crystallography including the complexes of IL-1R1/IL-1β, IL-1R1/IL-1Ra and IL-1R1/AF10847 (Fig. 1). While IL-1β is a cytokine that activates IL-1R, IL-1Ra is an IL-1 receptor antagonist and AF10847 is a peptide antagonist [20,44]. The crystal structure of IL-1R1/IL-1β (Fig. 1A) showed that three domains of IL-1R1 (D1-D3 domains) interact with IL-1β using extensive surface contacts. Their contact surface area (contributed from both proteins) is 4180.5 Å^2^ based on the structure of PDB entry: 4DEP calculated by NACCESS[45]. The interaction interface between IL-1R1/IL-1β with IL-1RAcP is less with a surface area of 2971.1 Å^2^. As reported, the antagonistic cytokine, IL-1Ra, interacts primarily with D1 and D2 domains of IL-1R1 but much less with the D3 domain of IL-1R1 (Fig. 1B)[19,28,46]. From our analysis, the D3 domain of IL-1R1 rotates 30 degrees away from that in the IL-1β bound IL-1R1 conformation; thus, making little contacts with IL-1Ra. The rotation of the D3 domain led to less contact interface area between IL-1R1 and IL-1Ra (3367.4 Å^2^). In both cytokine bound structures, the IL-1R1 ectodomain adopts a clamp form to bind with the spherical shape cytokine ligands that fold into a conserved 12 stranded beta sheet structure. Although the antagonistic helical peptide, AF10847, is much smaller in size (21 amino acids), a dramatic conformational change in the D3 domain of IL-1R1 facilitated via the loop between the D2 and D3 domains occurred and resulted in favorable interaction between IL-1R1 and AF10847 (Fig. 1C). Based on the structure of IL-1R1/AF10847, the contact interface area is 2505.4 Å^2^. The large interaction interfaces between IL-1R1 and different ligands also correlate with their high binding affinities. For examples, the IC50 values between IL-1Ra, AF10847 and IL-1R1 were reported to be 1.6 and 2.6 nM respectively [47]. Using a surface plasma resonance (SPR) based kinetic binding assay, the K_D_ values for IL-1R1 and IL-1β, IL-1Ra were reported to be 2.0 and 0.33 nM respectively [19]. Comparison of these three ligand-bound IL-1R1 structures (Fig. 1D) also suggested the loop between the D2 and D3 domains is much flexible than that between the D1 and D2 domains. This flexible loop permits the variation of the relative orientation between the D1-D2 domains and D3 domain and allows recognition of IL-1R1 to different ligands.

### Conformational sampling of IL-1R1 based on conventional and accelerated MD simulations

**Fig 2:**
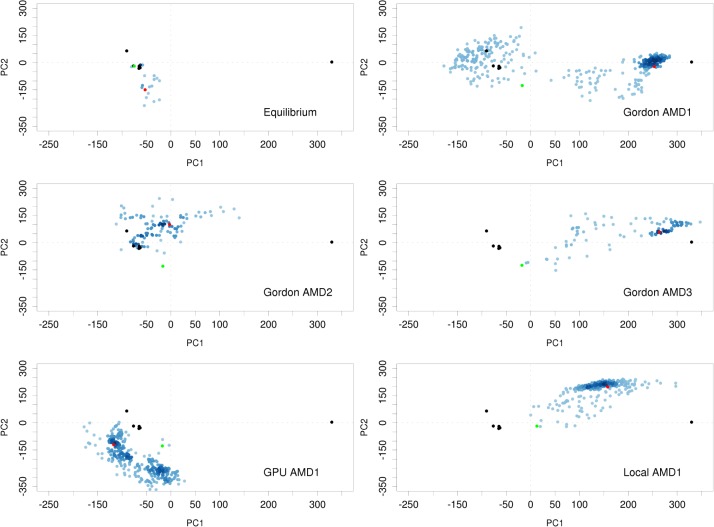
Mapping of IL-1R1 ectodomain conformations obtained from cMD and aMD simulations to the PC1 and PC2 subspace. Six crystal structures from five PDB entries: 4GAF, 1G0Y, 4DEP (2 chains), 1ITB and 1IRA (denoted by black circles), were used in the PCA to determine the first two principal components. Green and red circles denote the first and final conformations from each simulation.

The crystal structures of IL-1R1 with IL-1β, IL-1Ra and AF10847 gave static representations of IL-1R1 conformations stabilized by each ligand molecule. To explore and sample the conformational spaces of IL-1R1 extensively, we performed conventional and accelerated MD simulations of IL-1R1 using the IL-1R1 conformation in the IL-1R1/EBI-005 crystal structure. EBI-005 is an IL-1β chimeric protein that binds to IL-1R1 ectodomain at a higher affinity than IL-1β (K_D_ = < 0.014 versus 2.0 nM) [19]. To analyze the conformations of IL-1R1 obtained from the MD simulations, we first performed the principal component analysis (PCA) of the five available crystal structures of IL-1R1. Based on the PCA analysis, the first two principal components (PC1 and PC2) account for 98.6% of backbone conformational variations associated with the five crystal structures. As shown in Fig. 2, projections of the five IL-1R1 structures onto PC1 and PC2 subspace gave a clear distinction of the IL-1β- and IL-1Ra-bound IL-1R1 conformations from the AF10847-bound IL-1R1 conformation. Specifically, IL-1β-, IL-1Ra-bound IL-1R1 conformations were mapped to a similar region (PC1 = -70, PC2 = -20) whereas the AF10847-bound IL-1R1 conformation was projected to a further out region (PC1 = 330, PC2 = 4).

Although only five crystal structures of IL-1R1 in different ligand-bound conformations are available currently, the first two principal components of the PCA analysis can be informative to characterize the conformational changes of IL-1R1 in the MD simulations. Based on the mapping, conformations of IL-1R1 started with the EBI-005-bound conformation were found trapped in a region (PC1 = 140, PC2 = -80) in the 37 ns cMD run (S1 Fig.). IL-1R1 started with the AF10847-bound conformation remained confined at its initial conformational spaces during 20 ns cMD simulations (S1 Fig.). For the same 30 ns of simulation times, the IL-1Ra-bound IL-1R1 conformations overlap with part of the region visited by the EBI-005-bound IL-1R1 simulation. Results of cMD simulations indicated their limitation to overcome free energy barriers between different conformational microstates of IL-1R1 ectodomain because no overlapping conformations were found between EBI-005-bound and AF10847-bound IL-1R1 simulations.

In the aMD simulation, the potential energy wells were modified or raised to facilitate the barrier crossing of protein conformations between different microstates. Here, we used three different parameters denoted as AMD1, AMD2 and AMD3 where the modified potential wells become progressively shallower from AMD1 to AMD3. In Fig. 2B-D, we found the parameters used for AMD1 and AMD3 allowed sampling of IL-1R1 conformations to the proximity of the AF10847-bound IL-1R1 structure but not those using the AMD2 parameters. IL-1R1 conformations obtained from the GPU machine were found to map to different conformational spaces not accessed by other trajectories. Results from the aMD simulations indicated that the modifications to the potential energy wells permits extensive conformational sampling of IL-1R1 in a relatively short simulations times (118 ns in total). Projection of the IL-1R1 conformations from aMD simulations to the three dimensional subspace (PC1, PC2 and PC3) was provided in S2 Fig. which showed the extensive coverage of the conformational spaces including all ligand-bound conformations from the crystal structures.

**Fig 3:**
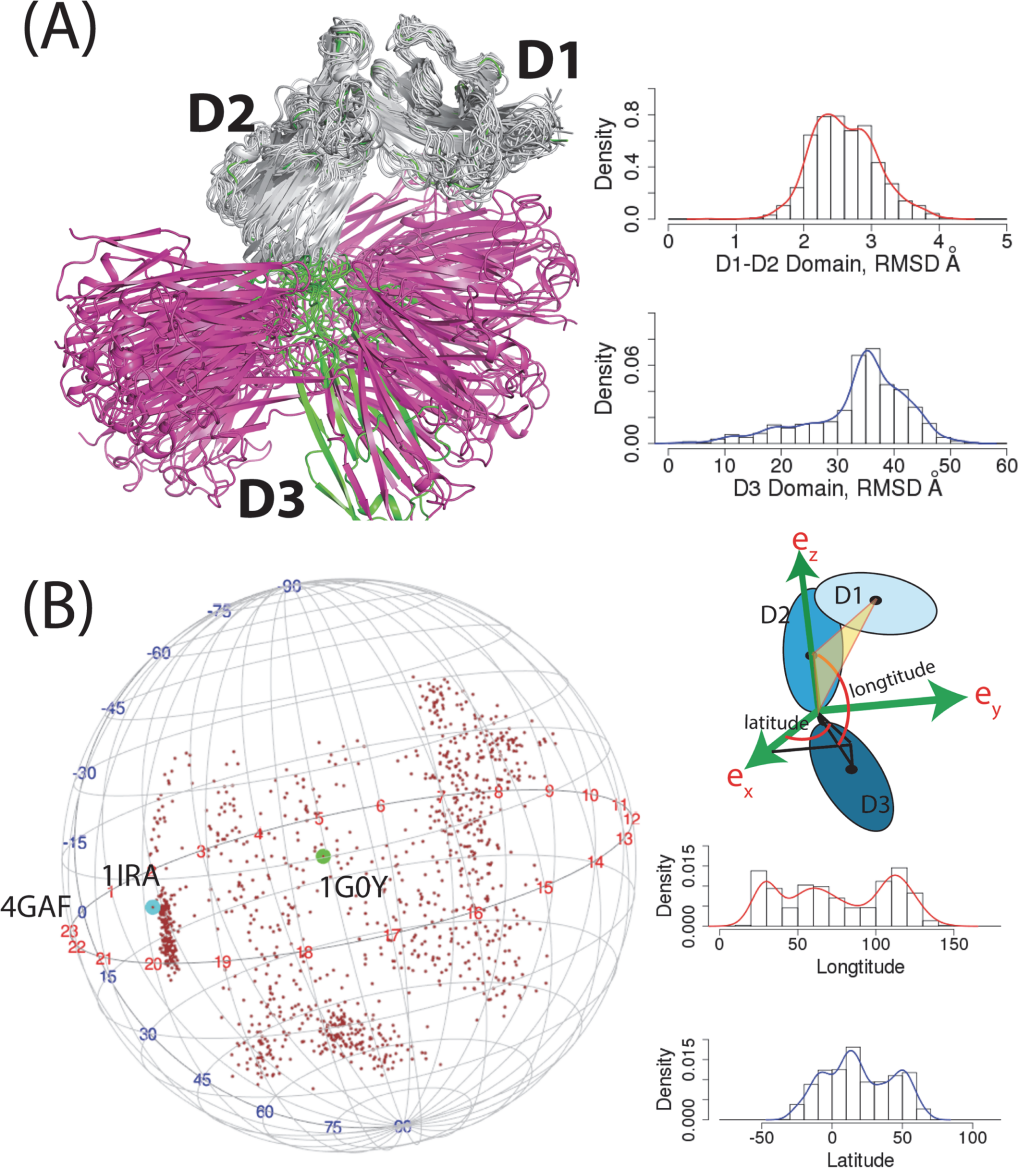
Superposition of 25 representative conformations obtained from the hierarchy cluster analysis and spherical projection of the center of mass of the D3 domain with respect to the D1-D2 domain. (A) The conformation colored in green is the IL-1R1 from the crystal structure (PDB entry: 4GAF). The D1-D2 domain was colored in grey while the D3 domain was colored in pink. All conformations were aligned to the D1-D2 domain of the crystal structure and the root-mean-square-deviations of the D1-D2 and the D3 domains were calculated accordingly. (B) Definition of the coordinate system was provided. Distributions of longitudinal and latitudinal angles were calculated based on the coordinate system. Conformations correspond to three crystal structures were labeled by their PDB entry names.

We further analyzed the conformational changes of IL-1R1 from the aMD simulations by studying backbone fluctuation of the D1, D2 and D3 domains. In all IL-1R1 conformations, the D1 and D2 domains of IL-1R1 showed very limited backbone variations where the root-mean-square deviations (RMSD) of the backbone atoms vary between 2–3 Å from the crystal structure (Fig. 3A). Larger backbone motions of IL-1R1 were observed in the D3 domain where the distribution of RMSDs peaks at 35 Å from the EBI-005-bound IL-1R1 crystal structure. The large RMSD values in the D3 domain do not result from the structural unfolding. It is attributed to the large rotation of the D3 domain anchored by the flexible loop between the D2 and D3 domains of IL-1R1. To characterize the rotational motion of the D3 relative to the D1-D2 domain, we defined a coordinate system as the following (Fig. 3B). The Cα atom of T217 at the C-terminus of the D2 domain is selected as the origin. The z-axis is defined as the vector connecting the origin and the center-of-mass of the D2 domain. The y-z plane is defined as the plane both the z-axis and the vector between the origin and the center-of-mass of the D1 domain lie on. Thus, the origin, the center-of-mass of the D1 and D2 domain are on the y-z plane. The x-axis is the axis perpendicular to the y-z plane. Relative orientation of the D3 domain to the D1-D2 domain is then represented by the vector connecting the origin and the center-of-mass of the D3 domain (Fig. 3B). Based on this coordinate system, the longitudinal motion of the D3 domain encompassed 110 degree (20 ─ 130) while the latitudinal motion covered a similar range of 100 degree (-30 ─ 70). The vectors from the origin to the center-of-mass of the D3 domain were mapped to a sphere shown in Fig. 3B to elucidate the large conformational variations of the D3 domain in the aMD simulation. Conformations of the IL-1Ra(1IRA)-, EBI-005(4GAF)- and AF10847(1G0Y)-bound IL-1R1 mapped on the sphere confirmed that they were included among those in the aMD simulations.

### Small molecule binding site analysis of IL-1R1 using Sitemap

**Fig 4:**
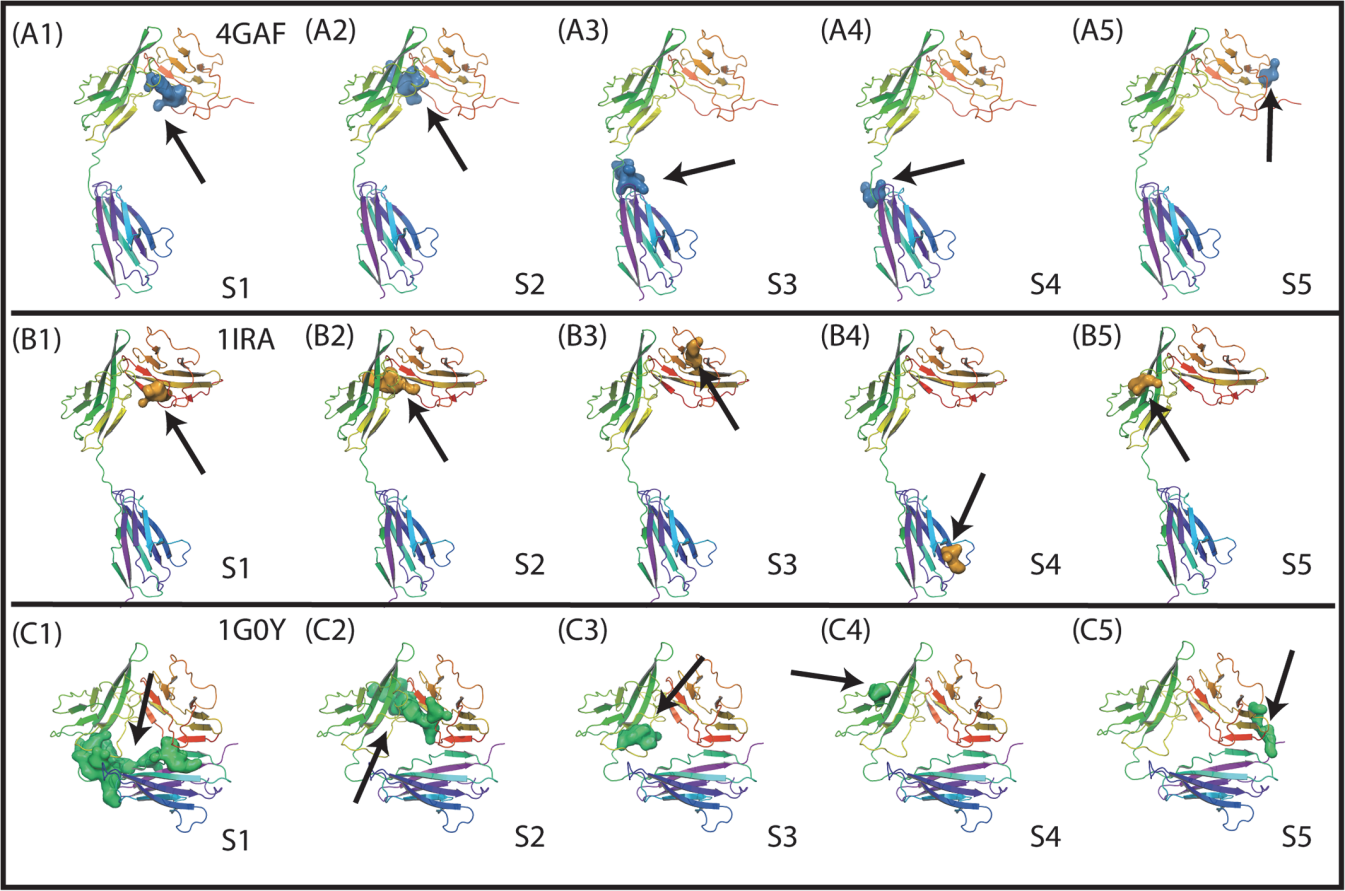
Small molecule binding site determined by the Sitemap analysis in three different IL-1R1 conformations extracted from the crystal structures. S1-S5 denote the binding site number in each conformation and the arrows point to their locations.

We first performed the Sitemap analysis to identify the small molecule binding sites in the crystal structures of three different ligand-bound IL-1R1 conformations. Two common binding sites were identified in the EBI-005- and IL-1Ra-bound IL-1R1 structures (cf. Fig. 4A1, 4A2, 4B1, 4B2). Both sites are located at the interface between D1 and D2 domains. A similar binding site at the same location was detected in the AF10847-bound IL-1R1 structure (Fig. 4C2). Two smaller binding sites close to the loop between D2 and D3 domains were detected in the EBI-005-bound IL-1R1 structure (Figs. 4A3 and 4A4). The top two ranked binding sites based on the Dscore values in the AF10847-bound IL-1R1 are much larger in sizes (volume: 738 and 439 respectively) than most other binding sites with the typical volume of less than 200 (Table 1). For inhibitor development consideration, small molecules bound to S1, S2 in EBI-005- and IL-1Ra-bound IL-1R1 conformations may directly inhibit IL-1R1 binding with the endogenous proteins that required the D1-D2 domains for high binding affinity such as IL-1Ra [28]. In contrast, small molecules bound to S3 and S4 of IL-1Ra can potentially impact on the relative orientation of D3 domain to the D1-D2 domains of IL-1R1. Because AF10847 antagonizes IL-1R1 by stabilizing IL-1R1 in an alternative conformation, small molecules bound to S1 of the AF10847-bound IL-1R1 may act as gluing molecules to trap Il-1R1 at this alternative conformation. To assess the potential of developing small molecule inhibitors targeting these sites, we used a druggability index discussed by Halgren’s Sitemap paper[22] which suggested undruggable binding sites have Dscore values < 0.83. Among the 15 binding sites detected as shown in Fig. 4, S1 Fig. and S2 Fig. are consensus druggable sites in IL-1R1 for small molecule inhibitors development according to the three crystal structures analyzed here (see Table 1). The S1 and S2 sites both have larger hydrophilic than hydrophobic surface area.

**Table 1:** Dscore values, volume sizes, hydrophilic and hydrophobic areas of the five binding sites in three IL-1R1 crystal structures (PDB entries: 4GAF, 1IRA and 1G0Y) identified by the Sitemap program.

	S1	S2	S3	S4	S5
Dscore					
4GAF	1.07	0.62	0.66	0.49	0.39
1IRA	0.71	0.93	0.55	0.54	0.47
1G0Y	0.94	1.00	0.74	0.54	0.47
Volume					
4GAF	162.58	114.56	160.52	61.06	61.07
1IRA	152.64	120.39	72.72	87.81	72.72
1G0Y	787.19	439.38	117.99	75.46	87.81
Hydrophilic area					
4GAF	738.00	560.00	891.00	406.00	462.00
1IRA	755.00	545.00	431.00	598.00	587.00
1G0Y	3970.00	3621.00	482.00	532.00	929.00
Hydrophobic area					
4GAF	116.00	43.00	25.00	16.00	10.00
1IRA	91.00	81.00	29.00	9.00	13.00
1G0Y	265.00	219.00	74.00	9.00	67.00

**Fig 5:**
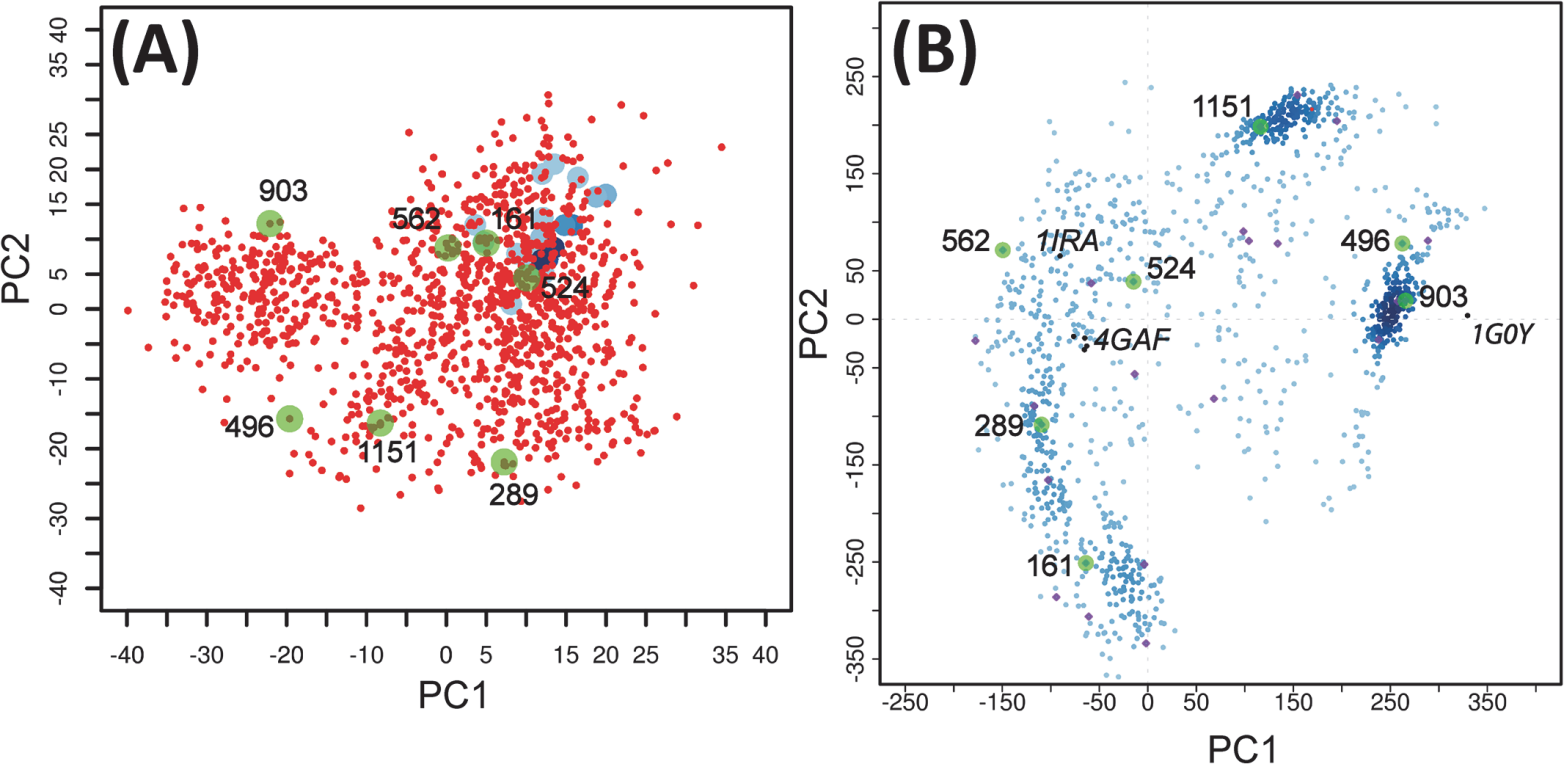
Mapping of IL-1R1 conformations to the first two principal components derived from two different PCAs. (A) PCA is based on the 1180 conformations generated from aMD simulations. Crystal structures were shown in blue circles. (B) The PCA is based on six crystal structures from five PDB entries: 4GAF, 1G0Y, 4DEP (2 chains), 1ITB and 1IRA (denoted by black circles). Representative conformations of the 25 cluster groups were shown in purple circles. Conformations discussed in the text were shown in green circles and labeled.

To investigate the small molecule binding sites in IL-1R1 conformations sampled by the aMD simulations, a manageable number of conformations are necessary for additional analyses. First, a total of 1180 conformations taken at every 100 ps from the combined 118 ns trajectory were selected ordered accordingly in the combined trajectory file. Characterization of the structural variations in the 1180 IL-1R1 conformations was analyzed by a second PCA of all 1180 conformations without including the crystal structures. Projection of the 1180 conformations to the first two PCs of this PCA were shown in Fig. 5A. In this PCA, the first two PCs accounted for only 40% of structural variations in all 1180 conformations and were insufficient to separate the crystal structures in distinct region. As a comparison, the projection of all 1180 conformations to PC1 and PC2 based on the PCA of the crystal structures was shown in Fig. 5B. To account for 90% of structural variations for conformational characterization, the first 38 PCs were included to construct the dendrogram in the hierarchy cluster analysis (see Methods). Second, 25 cluster groups were obtained by using a cutoff value of 40 Å at the dendrogram. Conformation at the center of each cluster group was used to represent each group whereas members in the same group have similar structural variations based on the cutoff values. Finally, the 25 representative conformations from each cluster group were used to represent the structurally different conformations obtained from the aMD simulation.

**Fig 6:**
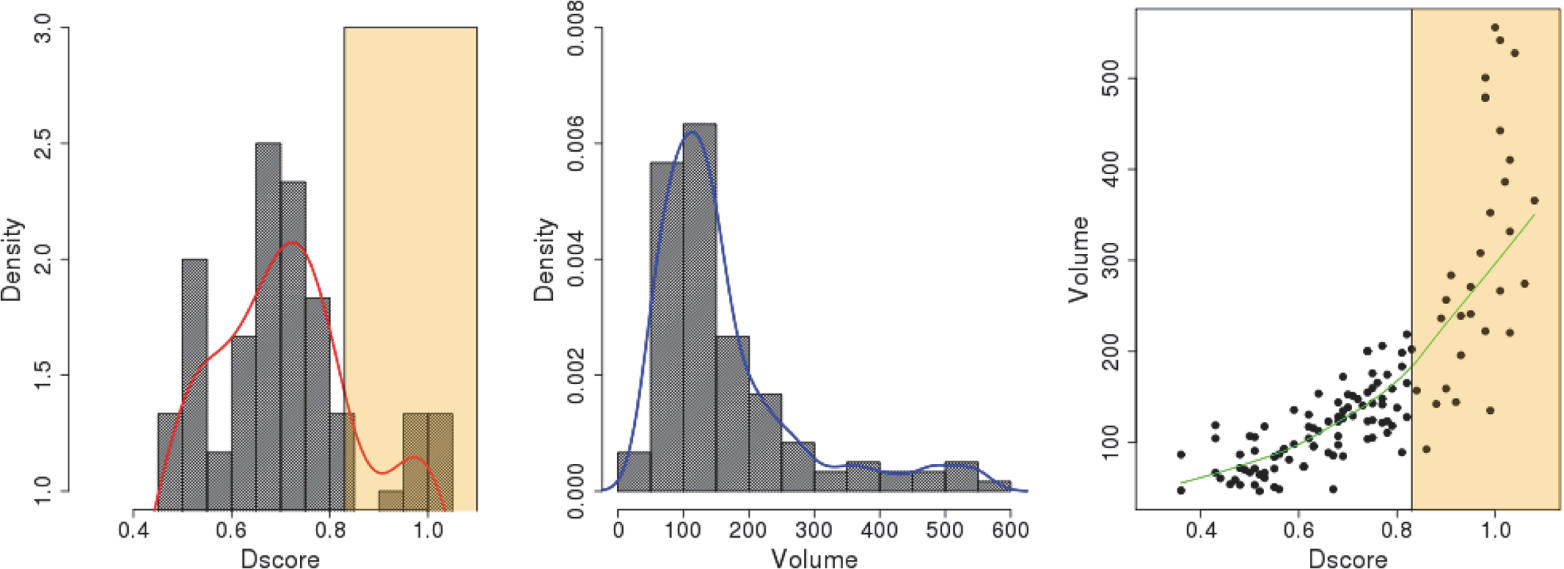
Distributions of Dscore values, volume sizes of the binding sites determined by the Sitemap analysis using 25 representative conformations obtained from the hierarchy cluster analysis. The vanilla color regions correspond to the binding sites with Dscore value ≥ 0.83.

Subsequent Sitemap analysis was performed on the 25 representative conformations to identify small molecule binding sites. As shown in Table 2, 32 of the 121 detected binding sites (27%) gave the Dscore ≥ 0.83 which is the same as those detected using the three crystal structures (27%). The distribution of the Dscore values for all binding sites was found to peak at 0.7 in Fig. 6. When analyzing the volume of each binding site, conformation **490** has a very large binding site with a volume of 1330 Å^3^ which is an outlier when compared with the sizes of the binding sites in all other conformations. The average volume of the druggable binding sites (Dscore ≥ 0.83) detected from the 25 representative conformations is 285.54 Å^3^ which is greater than those in the EBI-005- and IL-1Ra-bound IL-1R1 conformations (137.54 Å^3^). The differences of the binding site volumes are equivalent to a ligand of 34 versus 11 atoms. Although the peak of the volume distribution is at 150 Å^3^, an extended tail population between 300–500 Å^3^ can be found (Fig. 6). The volume sizes at the binding sites may be helpful for selecting appropriate sizes of compounds when building a library of compounds for screening. The Dscore value is also linearly proportional to the volume of the binding site although a steeper increase was seen after Dscore = 0.83 in Fig. 6. For the druggable binding sites with Dscore ≥ 0.83, the binding site volumes are around 200 Å^3^ and higher.

**Table 2:** Dscore values, Volume sizes of up to five binding sites identified by the Sitemap program in 25 representative IL-1R1 conformations determined by the hierarchy cluster analysis of 1180 aMD generated conformations.

Conf.	S1	S2	S3	S4	S5	Conf.	S1	S2	S3	S4	S5
**#**	**Dscore**					**#**	**Volume**				
**39**	**0.98**	0.71	0.43	0.56	0.53	**39**	**479.17**	151.26	104.62	48.71	61.06
**44**	**1.01**	0.77	0.50	0.53	0.55	**44**	**266.51**	206.14	67.23	117.65	51.11
**65**	0.78	0.68	0.56	0.53	0.52	**65**	123.48	144.06	87.12	66.89	46.65
**110**	0.82	**0.89**	0.59	0.74	0.51	**110**	165.33	**236.33**	135.83	200.31	71.34
**161**	0.57	**0.86**	0.69	0.58	0.55	**161**	92.61	**92.61**	172.19	81.29	71.34
**184**	**1.03**	0.75	0.69	0.68	0.55	**184**	**220.55**	105.30	134.80	97.41	84.38
**193**	0.66	0.79	0.70	0.68	0.52	**193**	88.84	159.15	152.64	128.28	64.83
**289**	**1.06**	**0.99**	0.59	0.72	0.48	**289**	**274.40**	**352.26**	98.10	147.83	86.78
**291**	**1.08**	**0.83**	0.67	0.70	0.62	**291**	**365.64**	**202.37**	85.75	138.57	117.31
**322**	0.50	**0.93**	0.82	0.64	0.51	**322**	107.02	**195.85**	127.94	153.66	90.90
**399**	0.49	0.81	0.71	0.36		**399**	69.97	198.60	129.31	47.33	
**450**	**0.95**	0.80	0.74	0.61	0.51	**450**	**241.13**	138.23	123.48	73.40	53.51
**490**	**0.95**	**0.94**	0.77			**490**	**270.89**	**1331.18**	148.18		
**496**	**0.98**	**0.92**	0.81	0.75	0.62	**496**	**500.44**	**144.40**	89.18	159.84	130.68
**524**	**1.03**	**0.91**	0.63	0.66	0.63	**524**	**331.68**	**283.66**	115.59	123.14	96.04
**562**	**0.90**	0.75	0.51	0.36	0.46	**562**	**159.15**	143.03	105.99	86.78	54.19
**674**	**0.93**	0.77	0.69	0.63	0.44	**674**	**239.07**	141.66	126.57	95.70	60.71
**787**	**1.00**	**0.98**	0.76	0.69	0.57	**787**	**555.66**	**478.14**	165.67	85.06	92.95
**854**	**1.04**	0.81	0.74	0.67		**854**	**527.53**	183.51	200.31	48.71	
**903**	**1.01**	0.82	**0.84**	0.68	0.61	**903**	**442.47**	218.83	**157.09**	123.14	73.75
**931**	**1.03**	0.68	0.43	0.48	0.47	**931**	**410.23**	107.02	119.02	53.51	59.00
**952**	**0.90**	**1.02**	0.77	0.75	0.74	**952**	**256.56**	**386.22**	121.77	124.51	155.04
**1006**	**0.97**	0.73	0.64	0.48	0.43	**1006**	**308.01**	140.63	112.85	72.03	66.89
**1015**	**1.01**	0.79	0.75	0.62	0.74	**1015**	**541.60**	118.34	175.96	104.62	103.93
**1151**	**0.99**	**0.88**	**0.98**	0.78	0.78	**1151**	**135.14**	**142.35**	**222.26**	174.59	110.79

Binding sites with the Dscore value ≥ 0.83 are shown in bold font.

**Fig 7:**
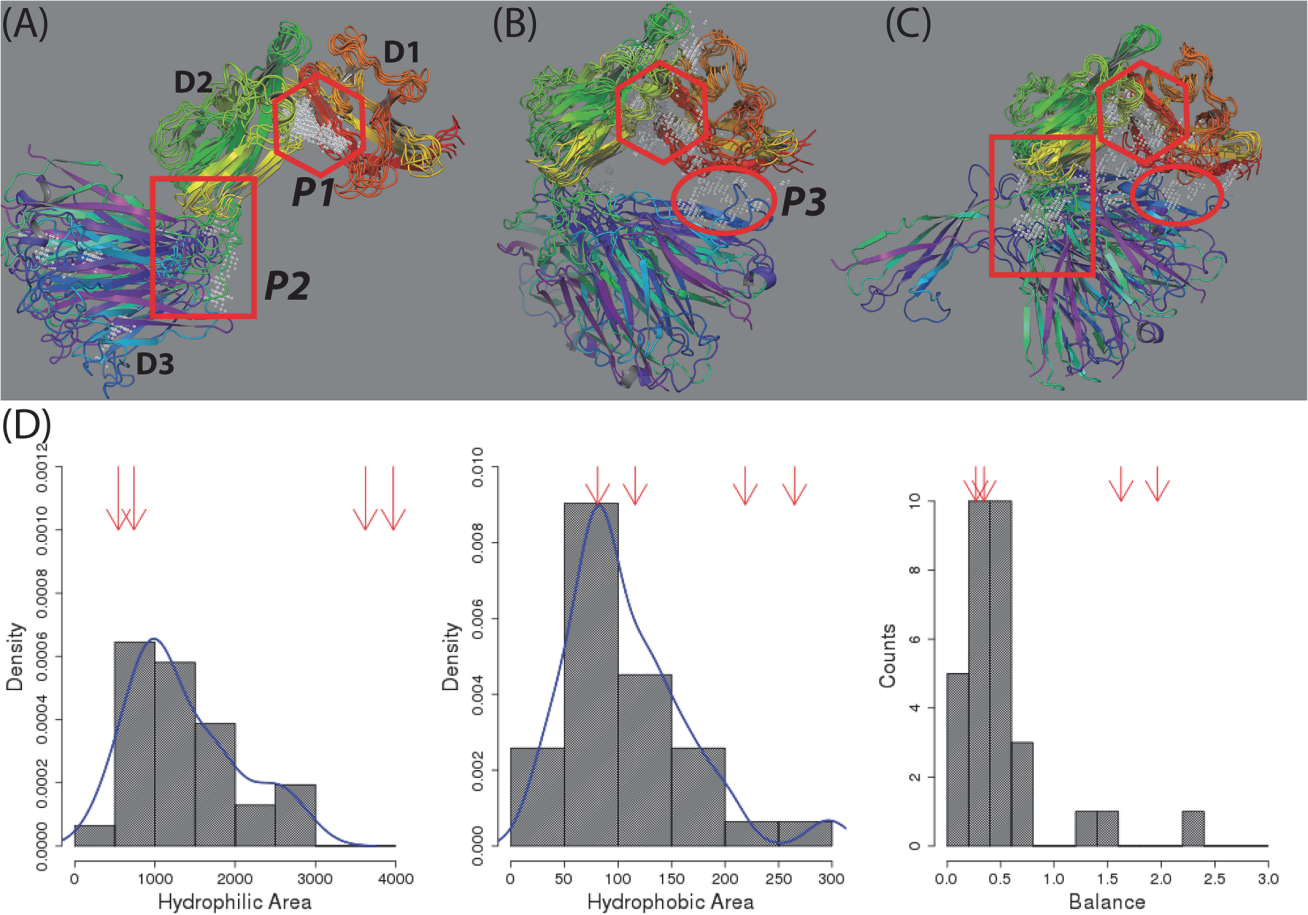
Locations of druggable sites in 25 representative conformations and their physicochemical properties determined by Sitemap. (A-C), druggable sites (Dscore values ≥ 0.83) in 25 representative conformations. Consensus binding sites at P1(diamond), P2(square) and P3(circle) locations in all conformations were shown. (D) Distributions of the physicochemical properties of the druggable sites. Balance refers to the ratio of hydrophobic and hydrophilic properties at the binding site. The four red arrows point to the values corresponding to four druggable sites in the crystal structures.

All probe points in the druggable sites of the 25 conformations from Sitemap were shown in grey color in three figures in Fig. 7. Most points were found clustered at three common regions (labeled P1, P2 and P3 in Fig. 7) which may be useful for developing inhibitors to prevent its binding with known ligands or small molecule modulators to affect the IL-1R1 ectodomain conformation (cf. Fig. 1). For example, P1 pocket can be used to develop inhibitors because it is located at the interface between the D1 and D2 domains which interact with IL-1β, IL-1Ra and AF10847. Small molecules binding to the P3 pocket located at the interface between the D1 and D3 domains may stabilize the inactive conformation of IL-1R1 similarly to that achieved by AF10847. The P2 pocket is located at the loop region between the D2 and D3 domains and was identified as binding sites in at least two different relative orientations of D3 to D2 domains as those shown in Figs. 4A3, 4A4 and 7A. Among the 25 conformations, those with the D3 domain orienting opposite to the D1-D2 domain (see Fig. 7A) have not been observed by experiments.

### Small molecule binding hotspot analysis of IL-1R1 based on the cosolvent mapping method

**Fig 8:**
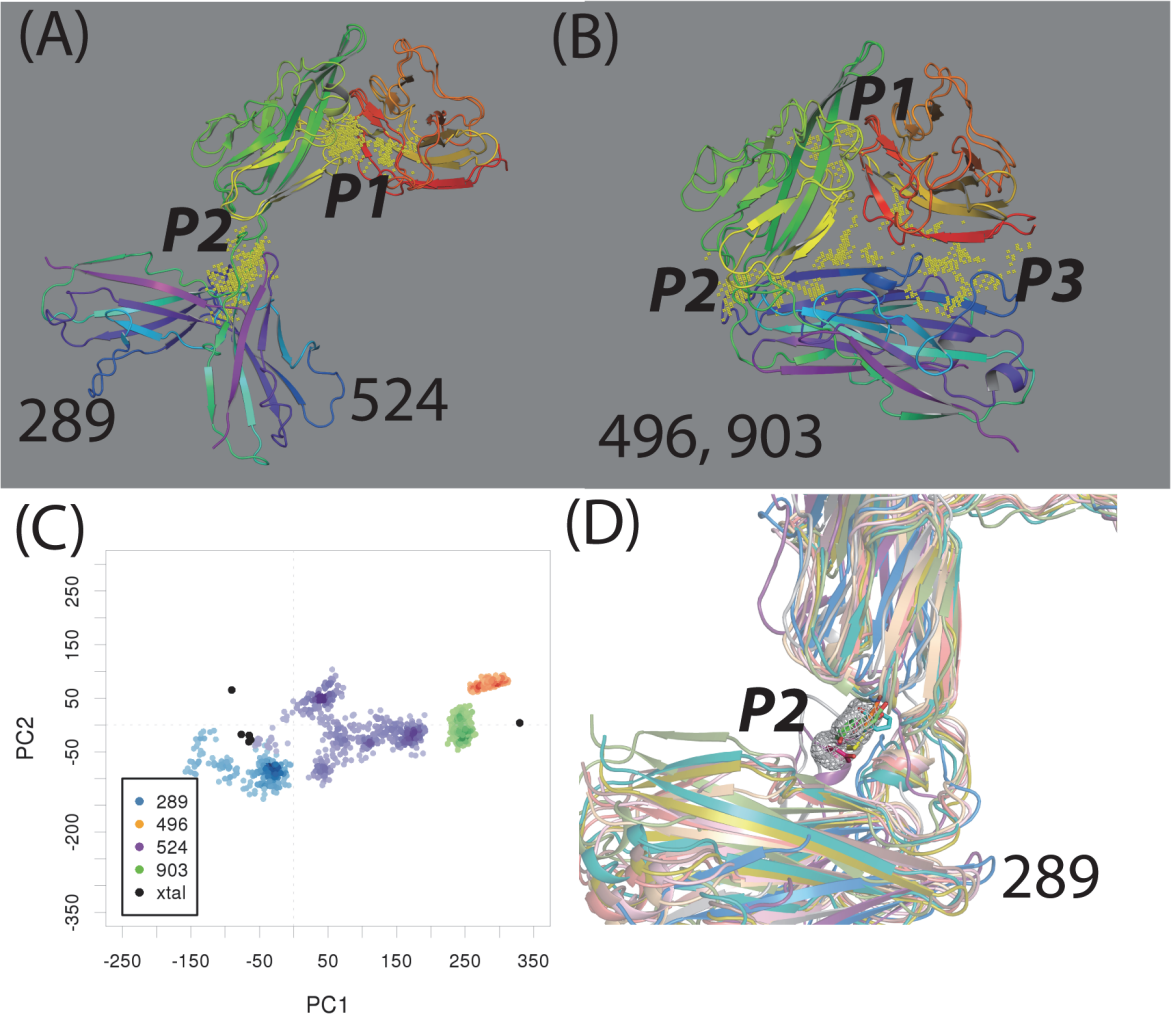
Druggble sites, mapping of conformations to PC1 and PC2 in conformations 289, 524, 496, and 903 and the small molecule hotspot at the P2 site of conformation 289. (A-B), druggable sites (yellow points) in conformations 289, 524, 496, and 903 identified by the Sitemap analysis. (C) Mapping of conformations obtained from the cosolvent MD simulations to the PC1-PC2 subspace. (D) The small molecule binding hotspot (the mesh envelop shape) at the P2 site in conformation 289 from the cosolvent mapping analysis. The phenol probe molecules within the mesh envelop region in 12 conformations from the cosolvent MD simulations were shown.

An emerging inhibitor development is to target potential binding sites not directly involved with protein-ligand binding (or allosteric binding sites) [48–50]. We investigated these types of binding sites in 32 druggable sites of 25 representative IL-1R1 conformations. Druggable sites were detected at the P2 and P3 sites in four conformations, i.e. **289**, **496**, **524** and **903** (Fig. 8A and 8B). While the P2 site was assessed druggable in conformations **289**, **496** and **903**, the P3 site were determined druggable in conformations **496** and **903** (see Fig. 8A, 8B and Table 2). To account for the binding site flexibility and its influence on the binding site druggability, we employed the cosolvent mapping method [23–25] for the binding hotspots assessment. Here, we used the phenol as the cosolvent molecule to probe these four IL-1R1 conformations because the phenyl group is frequently found in fragment screening library used to evaluate targets involved in protein-protein interaction[36].

Based on the 4 ns cosolvent MD simulations, we found that conformations **496** and **903** were stable during the simulations when their conformations were mapped to the PC1 and PC2 subspace (Fig. 8C). In contrast, conformations **289** and **524** underwent continuous conformational changes and deviated from their initial conformations. While conformation **289** quickly became trapped at a nearby region, conformation **524** wandered to a larger conformational subspace projected by PC1 and PC2 (Fig. 8C). The cosolvent mapping analysis further characterized the location of the hydrophobic hotspots preferentially bound by the phenyl molecules at the P1, P2 and P3 sites in these four conformations (Fig. 8D and S3 Fig.). Binding modes of phenol molecules at the P1 site in conformation **524** indicated greater flexibility of the P1 pocket as they interacted with IL-1R1 at multiple locations in the same region (S3 Fig.). For conformations **496** and **903**, less mobility of the phenol molecules at the P2 and P3 sites were found (S3 Fig.). For conformation **289**, the phenol molecules are found to be confined at smaller pockets in the P1 and P2 sites (Fig. 8D and S3 Fig.).

In the cosolvent MD simulations, the specific site found to interact with the probe molecules with less mobility reflects ideal complementary and can be considered as a hotspot region. Assessment of the results from cosolvent MD simulations indicated that the P2 site of conformation **289** and **903** and the P3 site of conformations **496** and **903** are binding hotspots using phenol as probes. The druggable property of the P2 site in conformation **524** was transient as the metastable conformation **524** deviated substantially from its initial conformation during the cosolvent MD simulation. The stability of each conformation was further evaluated by the 8 ns conventional MD simulations in which similar trends of conformational changes were observed (cf. Fig. 8C and S4 Fig.). Because conformation **289** represented a novel conformation of IL-1R1 not reported previously, we then focus on identifying candidate molecules that docked favorably into the P2 site of conformation **289** and assess their impacts on the IL-1R1 conformations.

### Targeting the allosteric modulator site of conformation 289 using *in silico* fragment library screening followed by MD simulations of the top eight ranked ligands with conformation 289

Based on the Sitemap evaluation and the cosolvent mapping analysis, the P2 site of conformation **289** warrants further investigation to determine how small molecules bound to the P2 site will affect IL-1R1 adopting the **289** conformation. Because the comparable size of P2 (352 Å^3^) to fragment compounds, we performed *in silico* screening to target the P2 site of conformation **289** using the Maybridge fragment library (a total of 2783 compounds). The Glide docking program was used to rank the predicted potencies of the compounds to the P2 site of conformation **289** based on their docked poses. The highest eight ranked compounds (denoted L154, L537, L951, L1192, L1206, L1882, L3097 and L3241) were selected and subject to additional 8 ns MD simulations to evaluate the effects of the ligand binding on IL-1R1 conformations.

**Fig 9:**
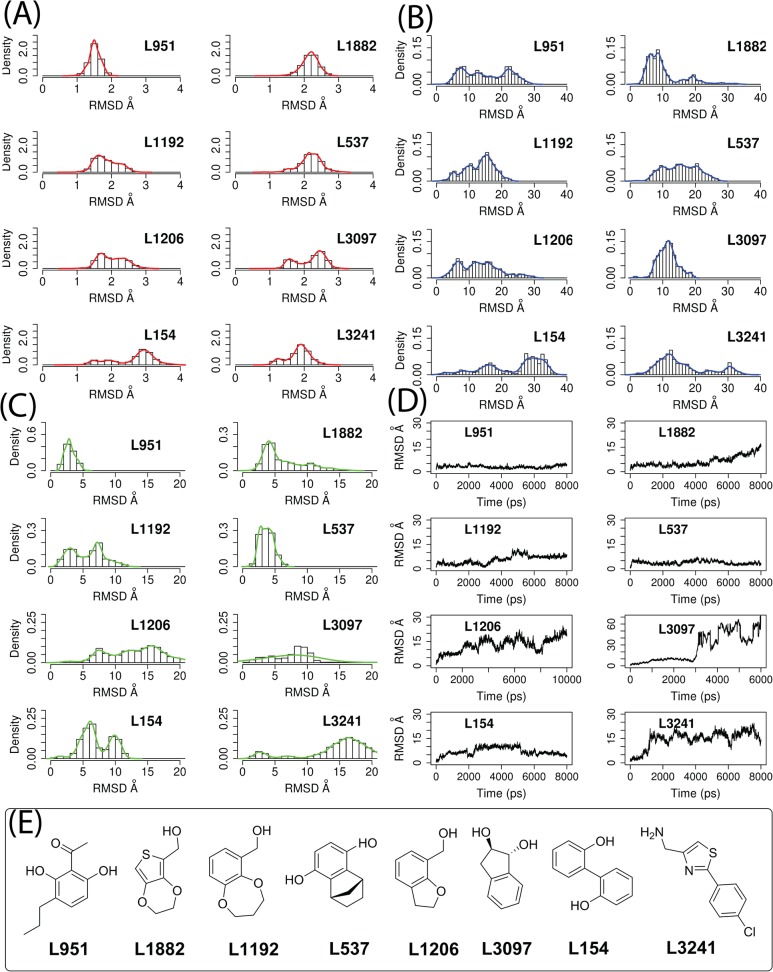
Distribution and trajectories of root-mean-square-deviations (RMSDs) of backbone atoms in the protein-ligand complexes from simulations. (A) is the D1-D2 domain, (B) the D3 domain and (C) heavy atoms of the ligands. The initial conformation 289/ligands conformations were used as the reference structures. Chemical structures of the eight ligands are provided in (D).

In Fig. 9, we calculated the root-mean-square deviations (RMSDs) of the backbone atoms in the D1-D2, D3 domains and the heavy atoms of the compounds in reference to the initial ligand poses and conformation **289** in the 8 ns MD simulations. All IL-1R1 conformations in the MD simulations were aligned to the D-D2 domain of the initial conformation **289**. Similar to the observation in the conformational sampling calculations, the D1-D2 domain in all eight fragment-bound IL-1R1 conformations exhibited less than 3 Å backbone deviations (Fig. 9A) except those with L154. The movement of the D3 domain is much greater even with the binding of these fragment ligands. The RMSD distributions of the D3 domain are less than 20 Å for L1192, L3097, L1206, 30 Å for L951, L1882, L537, and 40 Å for L154, L3241 (Fig. 9B). When monitoring the deviations of the ligands from their initial binding site locations, we found four ligands (L951, L1882, L1192 and L537) have major RMSD peak distributions at less than 5 Å whereas the other four (L1206, L3097, L154 and L3241) have broader distributions of RMSDs at greater than 5 Å (Fig. 9C). The RMSD values of L3097 along the trajectory (Fig. 9D) informed that L3097 escaped from the binding site. In contrast, L951 and L537 remained at the initial docked positions throughout 8 ns MD simulations (the peak distribution of RMSDs at around 2.5 Å). L1882 was close to its initial docked position only up to 4.8 ns whereas L1192 was up to 3 ns.

**Fig 10:**
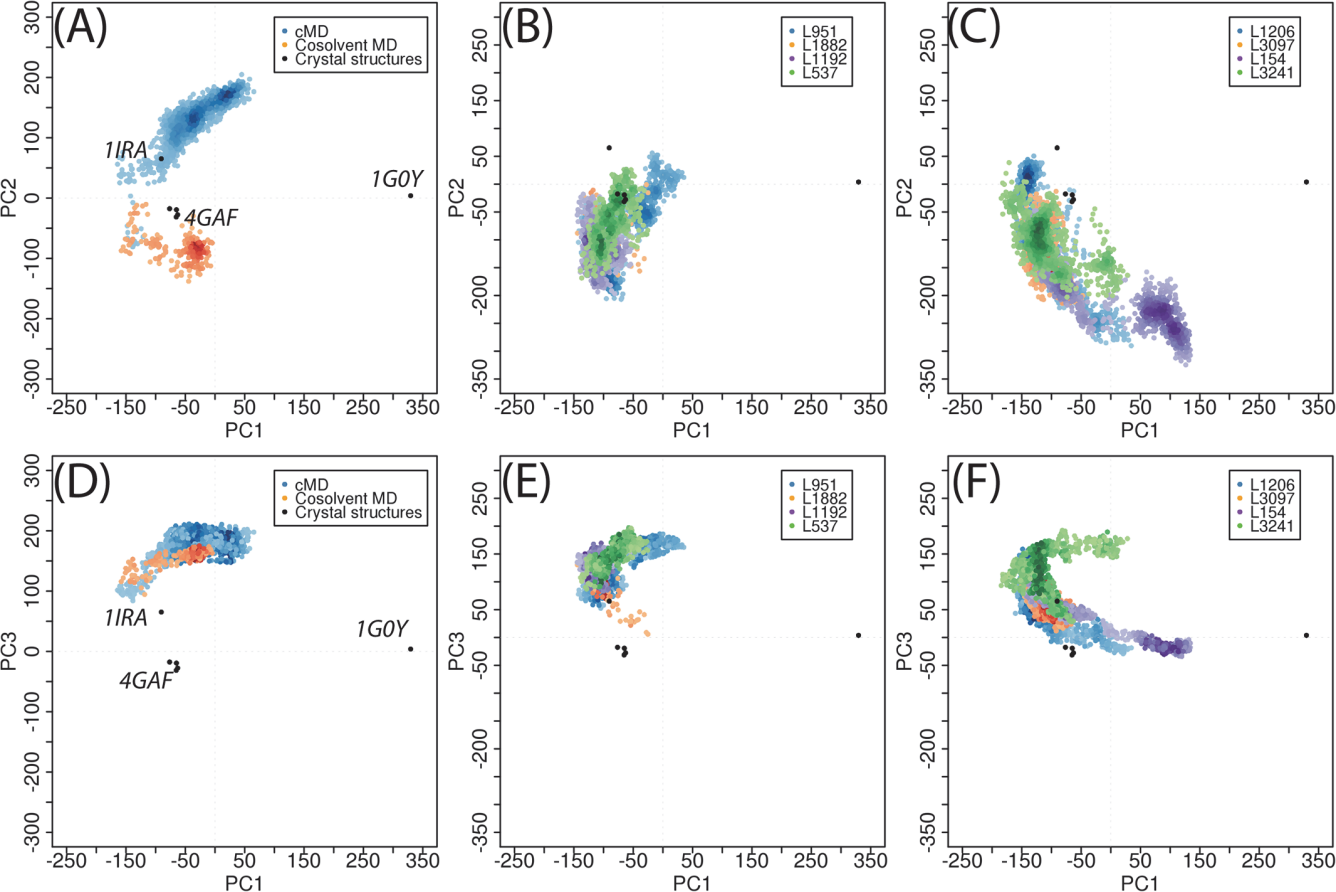
Mapping of IL-1R1 conformations obtained from different MD simulations to PC1/PC2 and PC1/PC3 subspaces. IL-1R1 conformations were extracted from 15 ns aqueous MD, 4 ns cosolvent MD simulations, L951/289, L1882/289, L1192/289, L537/289, L1206/289, L3097/289, L154/289 and L3241/289 MD simulations and mapped to PC1-PC2 (A-C) and PC1-PC3 (D-E) subspaces.

To investigate the conformational changes of these ligand-bound IL-1R1 conformations, we mapped them to the PC subspaces derived from the crystal structures. Two dimensional mapping to PC1/PC2 and PC1/PC3 were provided in Fig. 10. The mapping to PC1/PC3 subspace was provided because we found dynamical motion of IL-1R1 along the PC3 in the simulations. The results showed that IL-1R1 conformations obtained from four ligands (L951, L1882, L1192, L537) exhibiting smaller average RMSD values were projected to much localized regions in the PC1/PC2 and PC1/PC3 subspaces. In contrast, larger conformational spaces were visited by IL-1R1 bound with L1206, L154 and L3241. Although L3097 escaped from the docking site at around 0.9 ns, yet it caused IL-1R1 to trap at a local microstate. Based on the mapping of conformations to PC1 and PC2, the ligand-bound IL-1R1 conformations do not overlap with ligand-free IL-1R1 conformations but resembled those obtained from the cosolvent MD simulation.

**Fig 11:**
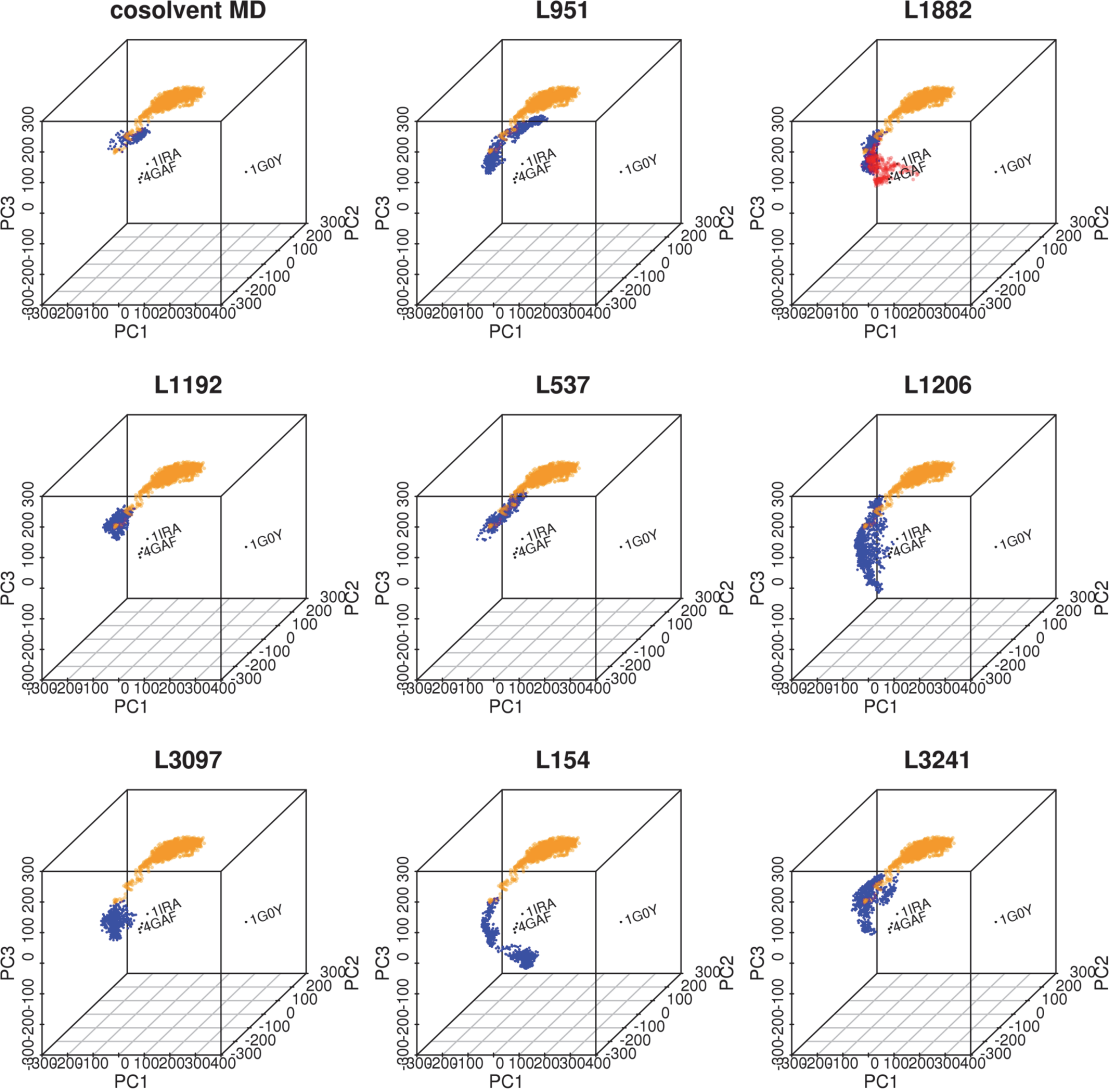
Mapping of IL-1R1 conformations (blue circles) obtained from the 4 ns cosolvent MD, 8 ns MD simulations of L951/289, L1882/289, L1192/289, L1206/289, L3097/289, L154/289 and L324/289 to PC1, PC2 and PC3. Trajectory of 15 ns MD simulations from the ligand-free conformation **289** was shown in orange circles. Trajectory of L1882/**289** after 4.8 ns was shown in red circles.

Two dimensional mapping in Fig. 10 implicated certain overlap of conformations between L951- and L537-bound IL-1R1 and IL-1β-bound IL-1R1. When we examined the IL-1R1 conformations, we found that they do not resemble the conformations of IL-1R1 conformations revealed by the crystal structures. This agrees with the large RMSD values of the D3 domain in IL-1R1 shown in Fig. 9 and the limitation of the two dimensional map. To elucidate the modulations of IL-1R1 conformations by these eight ligands, mapping of conformations to the three dimensional subspaces by PC1, PC2 and PC3 was used and shown in Fig. 11. IL-1R1 conformations were either locally trapped when bound with L1192 or transited along PC2 degree of freedom for L951 and L537. For L1882, IL-1R1 conformations were locally trapped for the first 4.8 ns and moved to the subspace closer to the IL-1β-bound conformation after L1882 escaped away from the binding site (Fig. 9D). Among the four other ligands, the initial interaction between L3097 and IL-1R1 caused the IL-1R1 to be trapped at a localized subspace even after L3097 dissociated from the P2 site. Broader conformational changes of IL-1R1 were found for the other three ligand-bound IL-1R1 conformations in which the ligands dissociated from the P2 site relatively early during the simulations. Ligands bound to the P2 site in IL-1R1 can potentially modulate the IL-1R1 conformations and impact on IL-1R1 binding with other protein ligands. Thus, L951, L1882, L1192 and L537 are attractive candidates for future experimental validation and evaluation.

**Fig 12:**
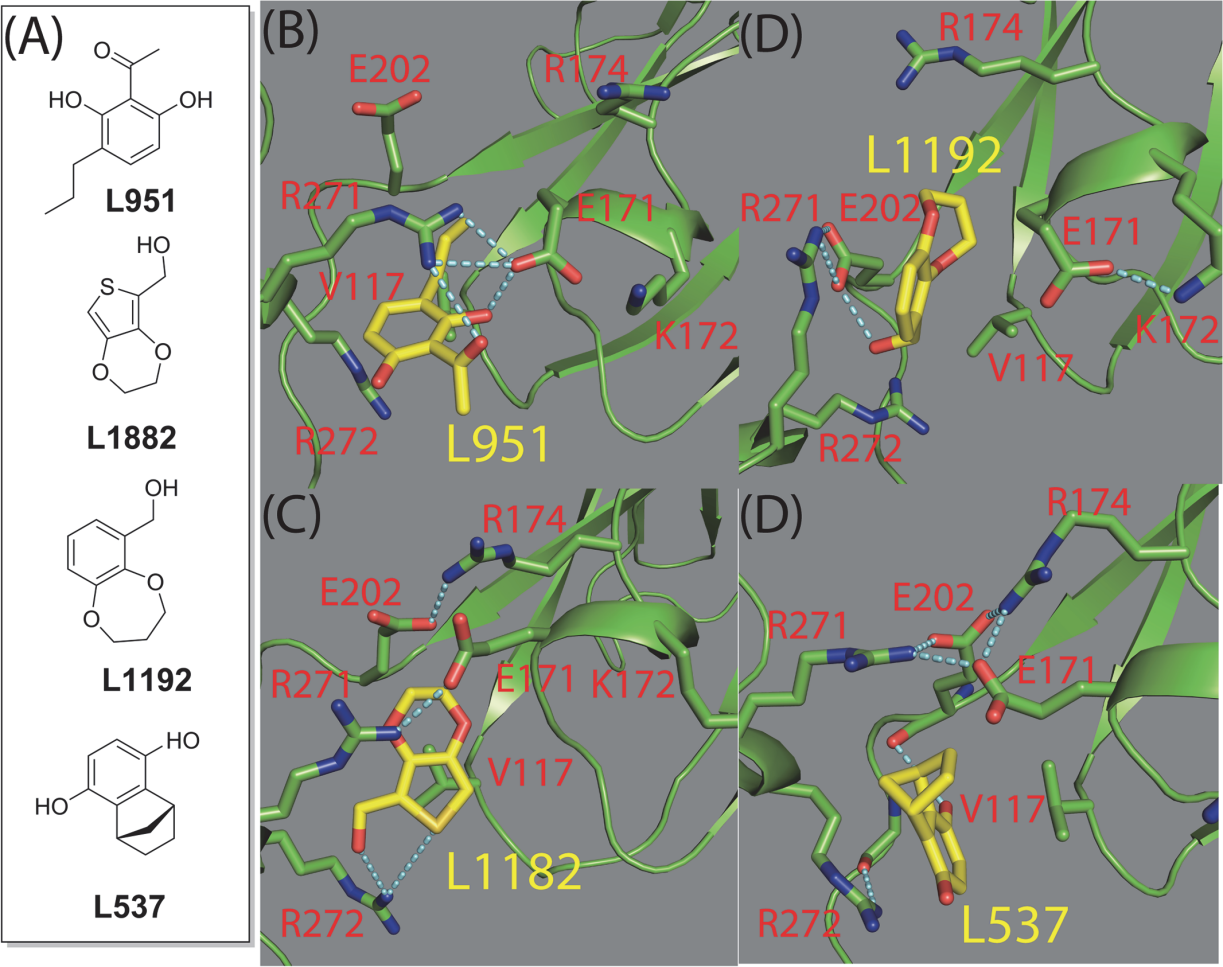
Chemical structures of four compounds identified from in silico screening to the P2 site in conformation 289 and snapshots of the IL-1R1/ligand conformations at 2 ns of MD simulations. L951, L1882, L1192 and L537 interact with IL-1R1 without significant deviations from their initial docked poses up to the 2 ns MD simulations. Residues in IL-1R1 interact with the ligands were shown in stick model and labelled. Hydrogen bonds were depicted in cyan dash lines.

Figs. 10 and 11 indicated that L951, L1882, L1192 and L537 bound IL-1R1 conformations underwent less conformational exploration in the PC subspaces (Figs 10 and 11). We further analyzed the protein-ligand poses of these four ligands at 2 ns of MD simulation where the ligands did not deviate much from their initial poses (RMSDs < 5) and interaction between IL-1R1 and ligands were established. We found a consensus binding site motif at the P2 site of IL-1R1 that interacts with the ligands via a similar type of protein-ligand interaction (Fig. 12). All four ligands interact with the binding pocket formed by R272, R271, V117, E202, E171, R174 and K172 between the D2 and D3 domains of IL-1R1. Salt-bridge interactions between Arg, Lys and Glu at the interface between the D2 and D3 domains establish a mall binding pocket for ligand binding. At the binding site, hydrogen bonds are formed between the hydroxyl groups of L951, L1882, L1192, L537 and E171, R272, E202 respectively. The carbonyl group of L951 and the thiophene group of L1882 form additional hydrogen bonds with R271 and R272. Furthermore, the aromatic rings of L951, L1192 and L537 interact with R271 and R272 via cation-aromatic interactions[51]. In this binding site, V117 is the key hydrophobic amino acid to interact with all ligands.

## Conclusion

Existing crystal structures of IL-1R1 with three different ligands including IL-1β, IL-1Ra and AF10847 indicated the large conformational flexibility of the loop between D1-D2 and D3 domains. We hypothesized the existence of transient small molecule binding sites among multiple conformational states accessed by IL-1R1 which may be suitable for inhibitor development. To identify these binding sites, we performed efficient conformational sampling of IL-1R1 using the accelerated MD algorithm. In a total of 118 ns aMD simulations started with a super cytokine (EBI-005)-bound IL-1R1 structure, we observed that the D3 domain of IL-1R1 exhibited a wide angular motion relative to the D1-D2 domain facilitated by the flexible loop between the D2 and D3 domains. These include the IL-1R1 conformations with close contacts between the D1-D2 and D3 domains mimicking closely to the AF10847-bound IL-1R1 conformation. In addition, we identified unreported IL-1R1 conformations with the D3 domain oriented to a position different from either IL-1Ra- or AF10847-bound IL-1R1 conformations.

Using the Sitemap analyses, we identified small molecule binding sites in the IL-1R1 conformations. The druggability index based on the Dscore value of these binding sites was then used to select “druggable” sites in these conformations for further evaluation. Four binding sites in three IL-1R1 crystal structures and 32 binding sites in 25 representative IL-1R1 conformations obtained from simulations gave the Dscore values ≥ 0.83 suggesting that they are potentially druggable. These druggable binding sites were found at three common locations in IL-1R1 including 1) the region between the D1 and D2 domain (the P1 Site), 2) the location between the D2 and D3 domains (the P2 site) and 3) the interface region between the D1 and D3 domain (the P3 site) when IL-1R1 adopts an inactive conformation.

Because the P1 and P3 sites can also be identified from existing crystal structures, we focused on investigating compounds potentially bound to the P2 site, an allosteric modulator site. Among the 25 representative conformations classified by the hierarchy cluster analysis, the P2 site in four conformations (**289**, **496**, **524** and **903**) gave Dscore values ≥ 0.83 indicating they are suitable for small molecule ligands binding. To account for the flexibility of the binding sites not included in the Sitemap analysis, we performed the cosolvent mapping analysis. Only the P2 site of conformation **289** and **903** were confirmed to be small molecule binding hotspots.

Because conformation **289** is a novel conformation not reported previously, an *in silico* screening by targeting the P2 site of conformation **289** was conducted using the Maybridge fragment library. Eight highest ranked ligands were selected and subjected to additional 8 ns conformation **289**/ligands MD simulations. Four ligands (L951, L1882, L1192 and L537) bound to the P2 site of conformation **289** for an extended period of time in the simulations and restricted the IL-1R1 conformations to a relatively localized region in the principal component subspaces. We further identified key residues in IL-1R1, including R271, R272, E202, E171 and V117, forming the binding pocket at the P2 site to interact with the ligands. The results suggested small molecule ligands can potentially bind to the P2 site of IL-1R1 and modulate the IL-1R1 conformations. Because conformation **289** differs from the IL-1R1 conformations observed in the crystal structures, potent chemical probes developed to target the P2 site of conformation **289** can potentially restrict IL-1R1 to an inactive conformation which abrogates the downstream IL-1 signaling. Additional *in vitro* experimental evaluation can validate our approach of discovering small molecule compounds targeting the allosteric modulator site in the ectodomain of IL-1R1. This strategy is attractive for the development of small molecule therapeutics targeting this difficult target that involved in protein-protein interaction and can potentially be applicable to other interleukin receptor proteins.

## Supporting Information

S1 Fig Projection to PC1 and PC2 subspaces using IL-1R1 conformations obtained from 37, 20 and 20 ns conventional MD simulations started with the EBI005, IL-1Ra and AF-10847 bound conformations. The green and red circles correspond to the initial and final conformations during the equilibrium production runs. The black circles correspond to the crystal structures.
(TIF)

S2 Fig Projection to PC1, PC2 and PC3 subspaces using IL-1R1 conformations obtained from the accelerated MD simulations. Crystal structures were show in black circles as reference.
(TIF)

S3 Fig Binding hotspots (mesh envelop shapes) and poses of the phenol probe molecules within the hotspots regions from the cosolvent MD simulations. Hotspots at the P1 sites of conformation 289 and 524 are shown in (A) and (B), at the P2 sites of conformations 289 and 903 are shown in (C) and (D), at the P3 sites of conformations 496 and 903 are shown in (E) and (F). All conformations were aligned the D1-D2 domains of the initial IL-1R1 conformations.
(TIF)

S4 Fig Projection to the PC1 and PC2 using conformations obtained from the 8 ns aqueous MD simulation of conformation 289, 496, 524 and 903. (TIF)
