# Supplementary figures and images for: Identification of Potential Small Molecule Allosteric Modulator Sites on IL-1R1 Ectodomain Using Accelerated Conformational Sampling Method
Source: PLoS One. 2015 Feb 23;10(2):e0118671. doi: 10.1371/journal.pone.0118671 (PMC4338101; doi:10.1371/journal.pone.0118671)

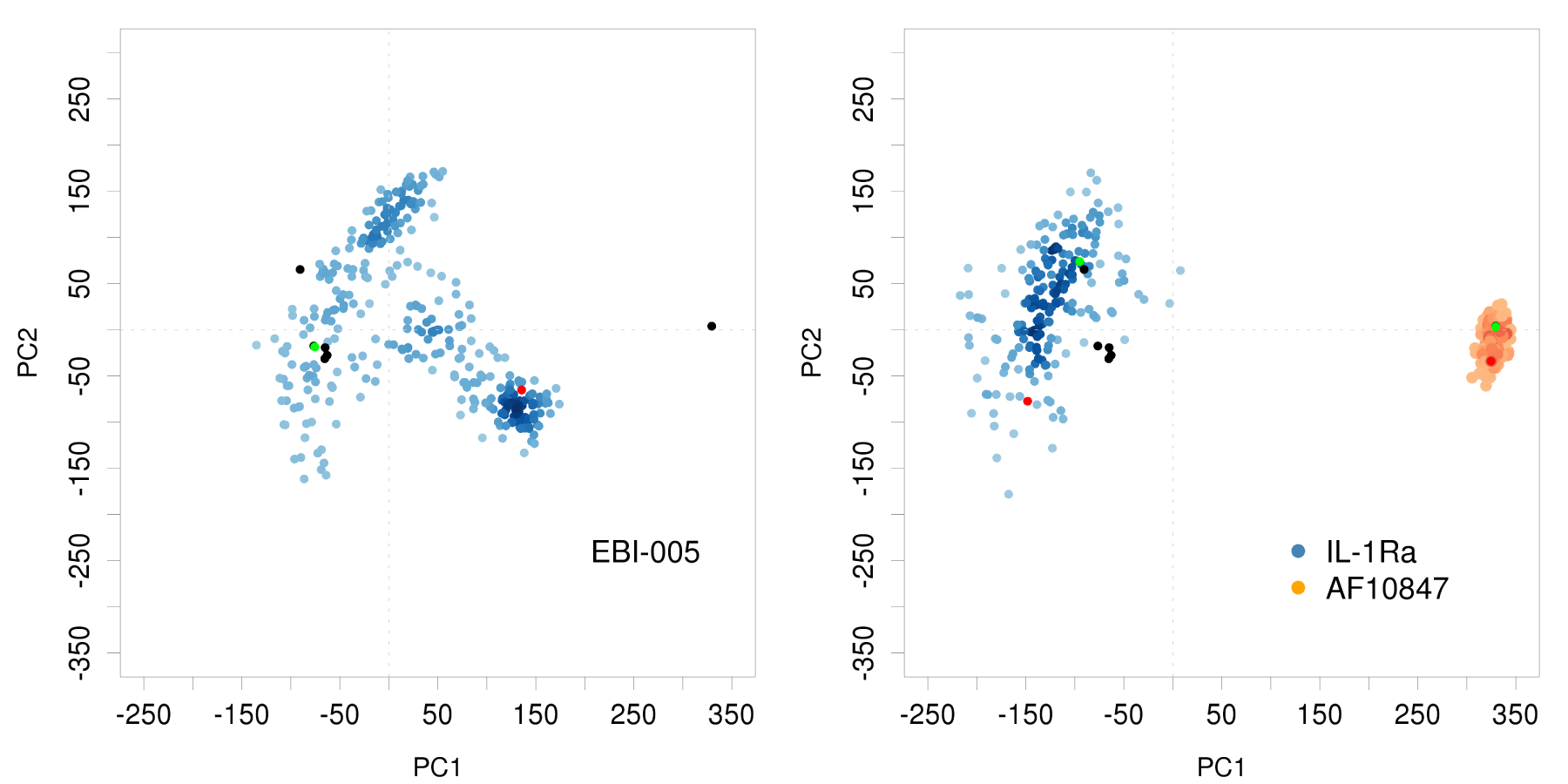

Supplement: S1 Fig — The green and red circles correspond to the initial and final conformations during the equilibrium production runs. The black circles correspond to the crystal structures. (TIF) [file pone.0118671.s001.tif]

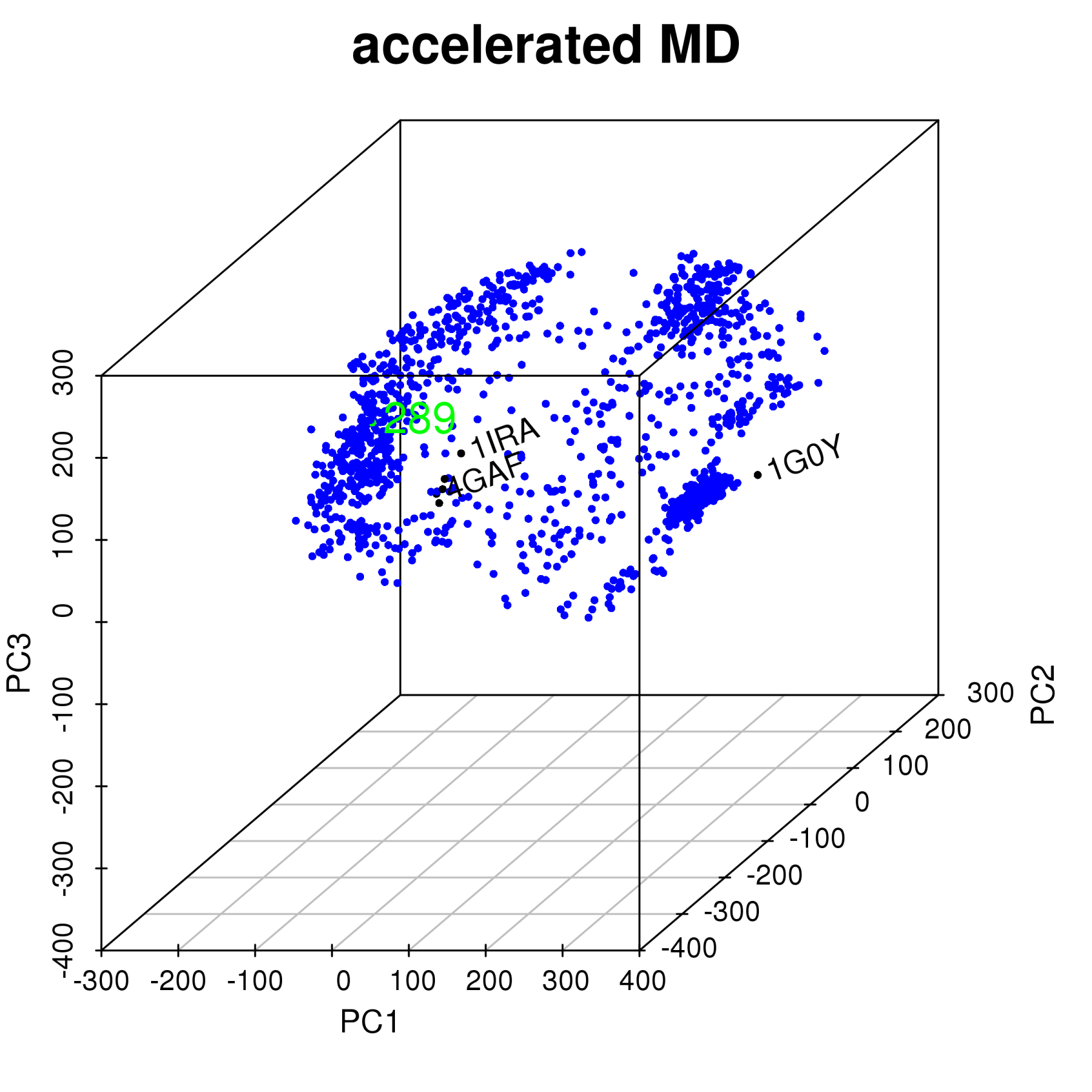

Supplement: S2 Fig — Crystal structures were show in black circles as reference. (TIF) [file pone.0118671.s002.tif]

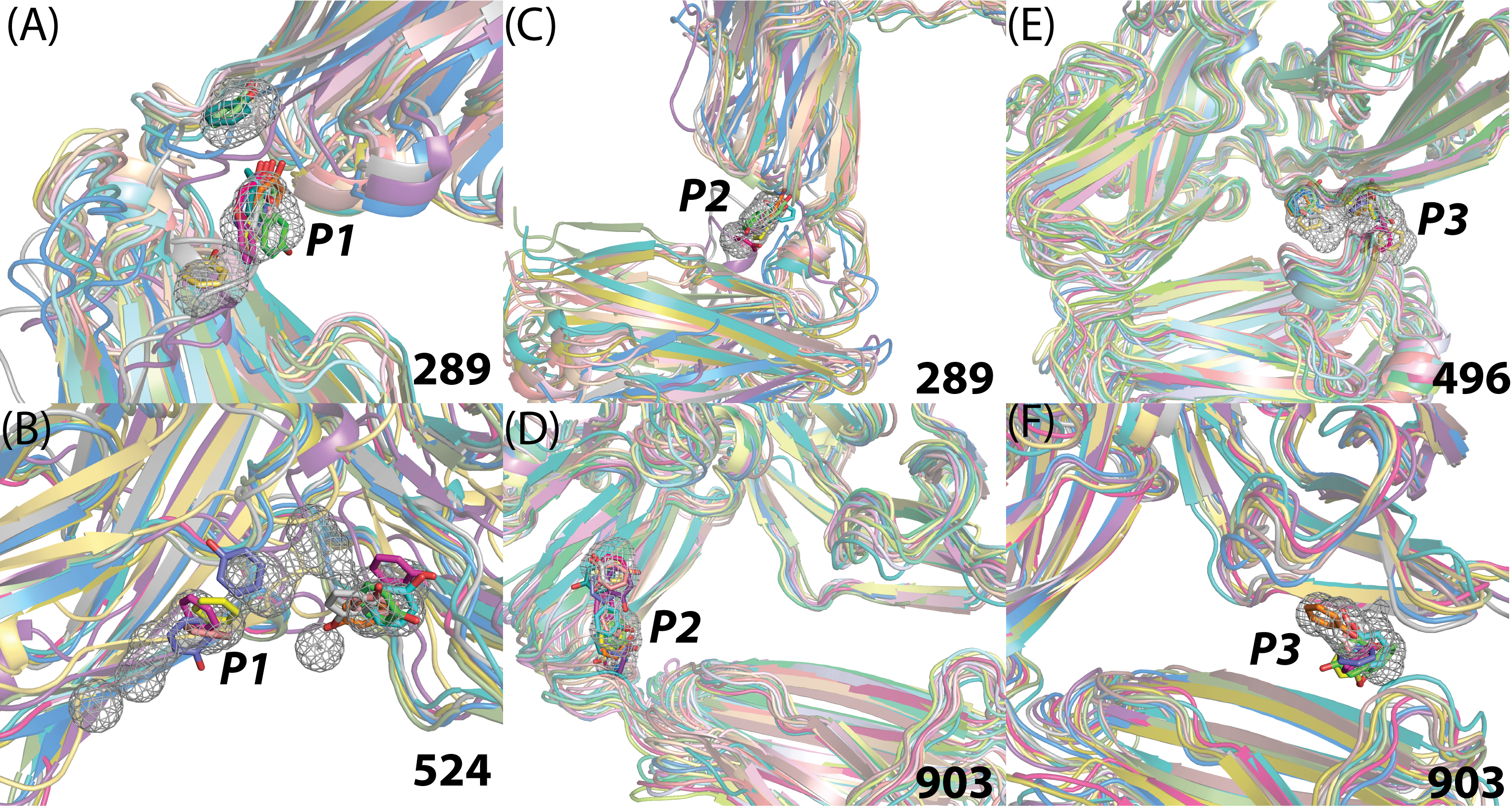

Supplement: S3 Fig — Hotspots at the P1 sites of conformation 289 and 524 are shown in (A) and (B), at the P2 sites of conformations 289 and 903 are shown in (C) and (D), at the P3 sites of conformations 496 and 903 are shown in (E) and (F). All conformations were aligned the D1-D2 domains of the initial IL-1R1 conformations. (TIF) [file pone.0118671.s003.tif]

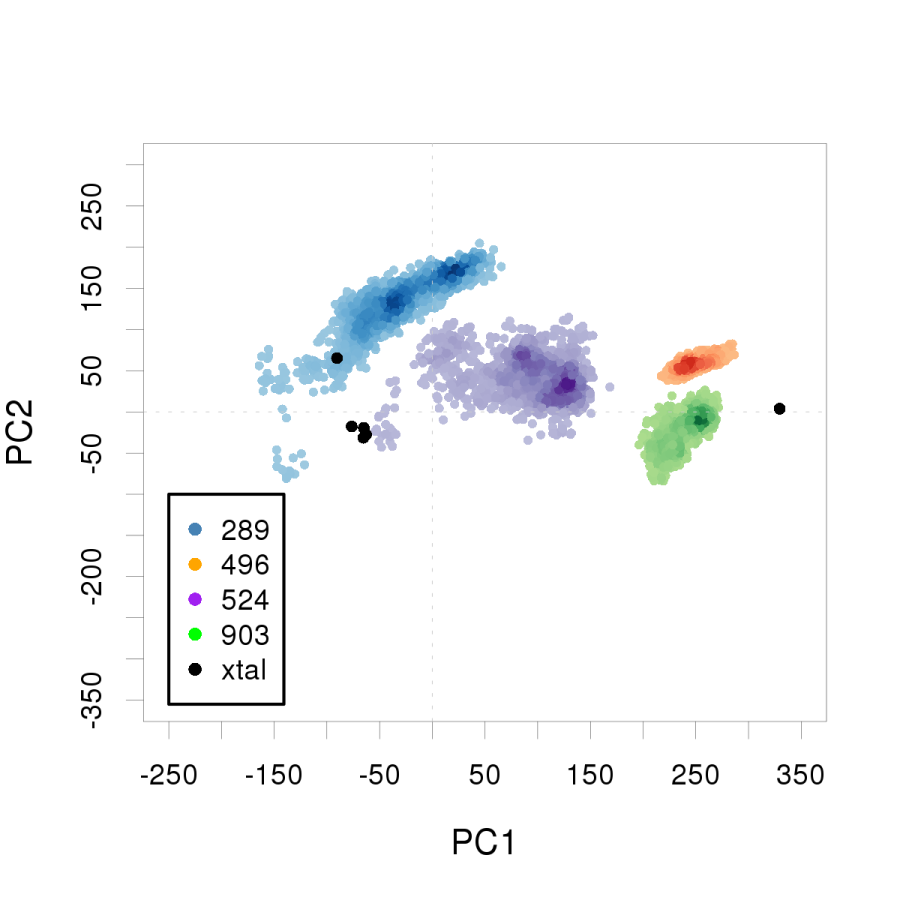

Supplement: S4 Fig — (TIF) [file pone.0118671.s004.tif]
